# P-769. A chromosome 4p14 locus associates with higher protection from Leprosy in close family contacts of Lepromatous Leprosy cases in endemic regions

**DOI:** 10.1093/ofid/ofae631.964

**Published:** 2025-01-29

**Authors:** Debdeep Mitra

**Affiliations:** Command Hospital Air Force Bangalore, Bangalore, Karnataka, India

## Abstract

**Background:**

Leprosy presents as an infectious disease with intricate genetic and immunological underpinnings. Genetic variations affecting cytokine genes and immune receptors, such as Toll-like receptor 1 (TLR1), may influence disease susceptibility. Close family contacts of individuals with slit skin positive lepromatous leprosy face a notably heightened risk of transmission. It is crucial to implement rigorous screening and early intervention protocols to promptly identify and manage latent infections, thereby curtailing potential disease dissemination within households and communities.

**Methods:**

We conducted a genome-wide association study involving close family contacts of individuals with positive slit skin smears for Mycobacterium leprae. Our study comprised 131 lepromatous leprosy patients from prospective cohorts in an endemic region. Genotyping was performed using real-time polymerase chain reaction (RT-PCR) on the 7500 Real Time PCR System, employing SNP genotyping assays for pre-selected SNPs (rs7842033 and rs4167826) with specific primers and probes corresponding to the SNP of interest.

**Results:**

Two-locus analyses of association of variants near rs7842033 showed a haplotype comprised of rs977477 and an IL9 missense variant, rs943138945, had the most significant association (p = 1.26x10-12).Our results showed no association between the SNP rs4052176 in NOD2 and leprosy in this population.

**Conclusion:**

Situated on chromosome four (4p14), the TLR1 gene encodes a receptor that identifies glycolipids and lipopolysaccharides of M. leprae, stimulating the transcription factor nuclear factor-kappa B (NF-kβ) and enhancing the expression of pro-inflammatory genes. This gene may confer a degree of protection to close contacts against Leprosy.

**Disclosures:**

**All Authors**: No reported disclosures

